# How and When to Use Lung Ultrasound in Patients with Heart Failure?

**DOI:** 10.31083/j.rcm2306198

**Published:** 2022-05-30

**Authors:** Stefano Coiro, Tripti Rastogi, Nicolas Girerd

**Affiliations:** ^1^Cardiology Department, Santa Maria della Misericordia Hospital, 06129 Perugia, Italy; ^2^Centre D'Investigation Clinique-Plurithématique Inserm CIC-P 1433, Inserm U1116, CHRU Nancy Hopitaux de Brabois, Institut Lorrain du Coeur et des Vaisseaux Louis Mathieu, F-CRIN INI-CRCT (Cardiovascular and Renal Clinical Trialists), Université de Lorraine, 54500 Vandoeuvre lès Nancy, France

**Keywords:** lung ultrasound, heart failure, cardiovascular diseases, cardiac oedema

## Abstract

Pulmonary congestion is a critical finding in patients with heart failure (HF) that can be quantified by lung ultrasound (LUS) through B-line quantification, the latter of which can be easily measured by all commercially-available probes/ultrasound equipment. As such, LUS represents a useful tool for the assessment of patients with both acute and chronic HF. Several imaging protocols have been described in the literature according to different clinical settings. While most studies have been performed with either the 8 or 28 chest zone protocol, the 28-zone protocol is more time-consuming while the 8-zone protocol offers the best trade-off with no sizeable loss of information. In the acute setting, LUS has excellent value in diagnosing acute HF, which is superior to physical examination and chest X-ray, particularly in instances of diagnostic uncertainty. In addition to its diagnostic value, accumulating evidence over the last decade (mainly derived from ambulatory settings or at discharge from an acute HF hospitalisation) suggests that LUS can also represent a useful prognostic tool for predicting adverse outcome in both HF with reduced (HFrEF) and preserved ejection fraction (HFpEF). It also allows real-time monitoring of pulmonary decongestion during treatment of acute HF. Additionally, LUS-guided therapy, when compared with usual care, has been shown to reduce the risk of HF hospitalisations at short- and mid-term follow-up. In addition, studies have shown good correlation between B-lines during exercise stress echocardiography and invasive, bio-humoral and echocardiographic indices of haemodynamic congestion; B-lines during exercise are also associated with worse prognosis in both HFrEF and HFpEF. Altogether, LUS represents a reliable and useful tool in the assessment of pulmonary congestion and risk stratification of HF patients throughout their entire journey (i.e., emergency department/acute settings, in-hospital management, discharge from acute HF hospitalisation, monitoring in the outpatient setting), with considerable diagnostic and prognostic implications.

## 1. Introduction

Signs and symptoms of congestion are a common cause of heart failure (HF) hospitalisation [1], even to a greater extent than the presence of clinical signs of hypoperfusion [2]. Gradual accumulation (within days) or rapid redistribution (within hours) of intravascular and interstitial fluids [i.e., extravascular lung water (EVLW)] are the main causes of the two most common clinical presentations of acute HF (AHF), namely decompensated HF and pulmonary oedema (PO), respectively [2, 3]. Of note, signs and symptoms of pulmonary congestion are the most common findings in AHF, encountered in approximately 75% of AHF patients [2, 4].

Lung ultrasound (LUS) imaging has emerged as a simple semi-quantitative method to detect and assess pulmonary congestion in HF patients through the quantification of B-lines (also known as “comet-tail artifacts” or “lung comets” prior to the release of an International Consensus) [5]. These B-lines are reverberation artifacts, originating from water-thickened pulmonary interlobular septa [6, 7, 8, 9].

The diagnostic usefulness of B-lines has been initially identified in the intensive care unit (ICU) to differentiate PO from other causes of acute respiratory distress syndrome (ARDS) [10]. In this setting, B-lines were closely related to thickenings of the sub-pleural interlobular septa and ground-glass areas assessed by computed tomography [10]. Subsequently, LUS has demonstrated its diagnostic value in identifying a cardiogenic origin of dyspnoea in various settings [i.e., pre-hospital, emergency department (ED), ICU, cardiology/inpatient units] [10, 11, 12, 13, 14, 15, 16, 17]. Additionally, increasing evidence supports that LUS has a sizeable prognostic value in patients with AHF [18], both on admission [19, 20], and at discharge [21, 22]. Furthermore, dynamic changes in B-lines have been promoted as an efficient monitoring of pulmonary decongestion to assess diuretic response [23, 24].

Over the course of the last decade, accumulating evidence derived from ambulatory settings has suggested that LUS, in addition to its potent diagnostic value [25, 26, 27], also represents a key prognostic tool in both HF with reduced (HFrEF) and preserved ejection fraction (HFpEF) [25, 27, 28, 29]. Furthermore, LUS-guided therapy, as compared with standard care, has been shown to reduce the short- and mid-term risk of HF hospitalisations [30, 31]. Finally, B-line changes during exercise stress echocardiography (ESE) were shown to be correlated with different indices of haemodynamic congestion, and also associated with worse prognosis in both HFrEF and HFpEF [32, 33, 34, 35, 36].

The present review summarises current evidence on the use of LUS methodology, its applications as well findings in patients with HF in various clinical settings.

## 2. Lung Ultrasound Methodology

### 2.1 Imaging Transducers

LUS examination can be performed using any commercially available 2-D echocardiographic equipment, and with any transducer probe (i.e., phased-array probes, high frequency linear-probes, curvilinear probes). Each transducer has specific advantages and disadvantages: namely, phased-array probes, as compared with curvilinear probes, have a multipurpose use by allowing both LUS and cardiac examinations; conversely, the footprint size of curvilinear probes (i.e., the length of the active transducer face in contact with the skin) render scanning between ribs more challenging comparatively to phased-array probes [37]. B-lines can be detected by all probes, although low frequency probes (i.e., phased-array or curvilinear probes in the 1 to 5 MHz range) are likely the most suitable for this purpose [38]. While B-line counts may slightly differ when using different transducers in a specific chest zone, the overall clinical picture is not affected by the use of a particular probe [38].

### 2.2 Lung Ultrasound: Normal and Interstitial Patterns

In normal conditions, the only displayable structure is the pleura, a hyperechoic horizontal line which moves synchronously with respiration; this movement is called lung sliding [38]. Additionally, there are some hyperechoic horizontal lines arising at regular intervals from the pleural line: the A-lines. These two findings represent the “A-profile”, a sign of normal content of air in the alveolar spaces. It can be encountered in patients with pneumothorax, asthma or pulmonary embolism [13].

Both physical examination and chest radiography are affected by low sensitivity (about 50–60%) in diagnosing pulmonary congestion in AHF [39]. LUS enables the detection of pulmonary congestion in patients presenting with acute dyspnoea with higher accuracy than chest auscultation or chest X-ray [17]. B-lines, the sonographic sign of alveolar-interstitial syndrome, increase together with a decrease in lung air content, resulting from an impedance mismatch between air and fluid-filled interlobular septa [10].

In the context of AHF, B-lines are comet tail (vertical) hyperechoic artifacts which arise from the pleural line, moving synchronously with lung sliding. They are virtually constantly well-defined and laser-like, extending downward to the edge of the screen, often erasing A-lines [40]. Two other vertical artifacts, not fulfilling the aforementioned criteria, can be displayed by LUS: Z-lines (short, not erasing A-lines; no pathologic significance), and E-lines (arising above the pleural line in subcutaneous emphysema) [41].

The presence of >2 B-lines in a single chest zone space is termed the “B-profile”, which is suggestive of an alveolar or interstitial process, including PO, ARDS or pulmonary fibrosis [5]; differential diagnosis requires additional clinical and sonographic information.

With regard to LUS findings, B-lines are dynamic in the setting of pulmonary congestion associated with AHF, and can resolve rapidly with treatment [37]. B-line quantification has been generally reported as a count-based method (i.e., the sum of B-lines recorded at each scanning site) or as a scoring system (i.e., the number of “positive zones”, defined as a minimum number of B-lines in one scanning site). According to current recommendations, ≥2 positive zones (a positive zone requires at least 3 B-lines) on both sides are consistent with a diagnosis of AHF [5, 42]. However, when comparing different LUS techniques, 1 or more positive zones bilaterally using an 8-zone method was actually found to have the maximum diagnostic value for AHF in patients with acute dyspnoea [43]. With regards to the count-based method, Picano *et al*. [11] proposed a B-line grading for the 28-zone method as follows: mild (6–15 B-lines), moderate (16–30 B-lines), and severe (>30 B-lines) pulmonary congestion. A similar count-based method can be applied to the 8-zone method: a threshold of 3 B-lines has been shown to have sizeable prognostic value both in hospitalised and ambulatory patients.

In the setting of acute dyspnoea, it may be challenging to differentiate PO from ARDS. As opposed to PO, inhomogeneous interstitial pattern (the “A/B profile”, i.e., predominant B-profile on one side and predominant A profile on the other), highly fragmented pleural line, reduced/abolished lung sliding, and presence of lung consolidation are commonly encountered in ARDS [13, 38, 40]. Similarly, LUS findings of COVID-19 pneumonia are similar to those observed in ARDS [44]. B-lines are frequently seen but may have a patchy distribution, the pleural line may appear irregular with areas of discontinuity; as disease severity progresses, small subpleural hypoechoic consolidations appear.

### 2.3 Scanning Technique

The transducer can be placed perpendicular to the ribs with the indicator facing cephalad (defined as longitudinal or sagittal scans) or, alternatively, positioned parallel to the intercostal space (defined as oblique or transverse scans) in order to display a larger section of the pleura [37, 38]. LUS can be performed with the patient in any position. However, patient positioning should be standardised when performing serial lung ultrasonographies due to its impact on B-line counts since patients with AHF may have a greater number of B-lines in the supine compared with the sitting position [45]. B-line variability can also occur according to the number of zones, echocardiographic equipment, clip-length, and type of transducer [46].

**Fig. 1.**
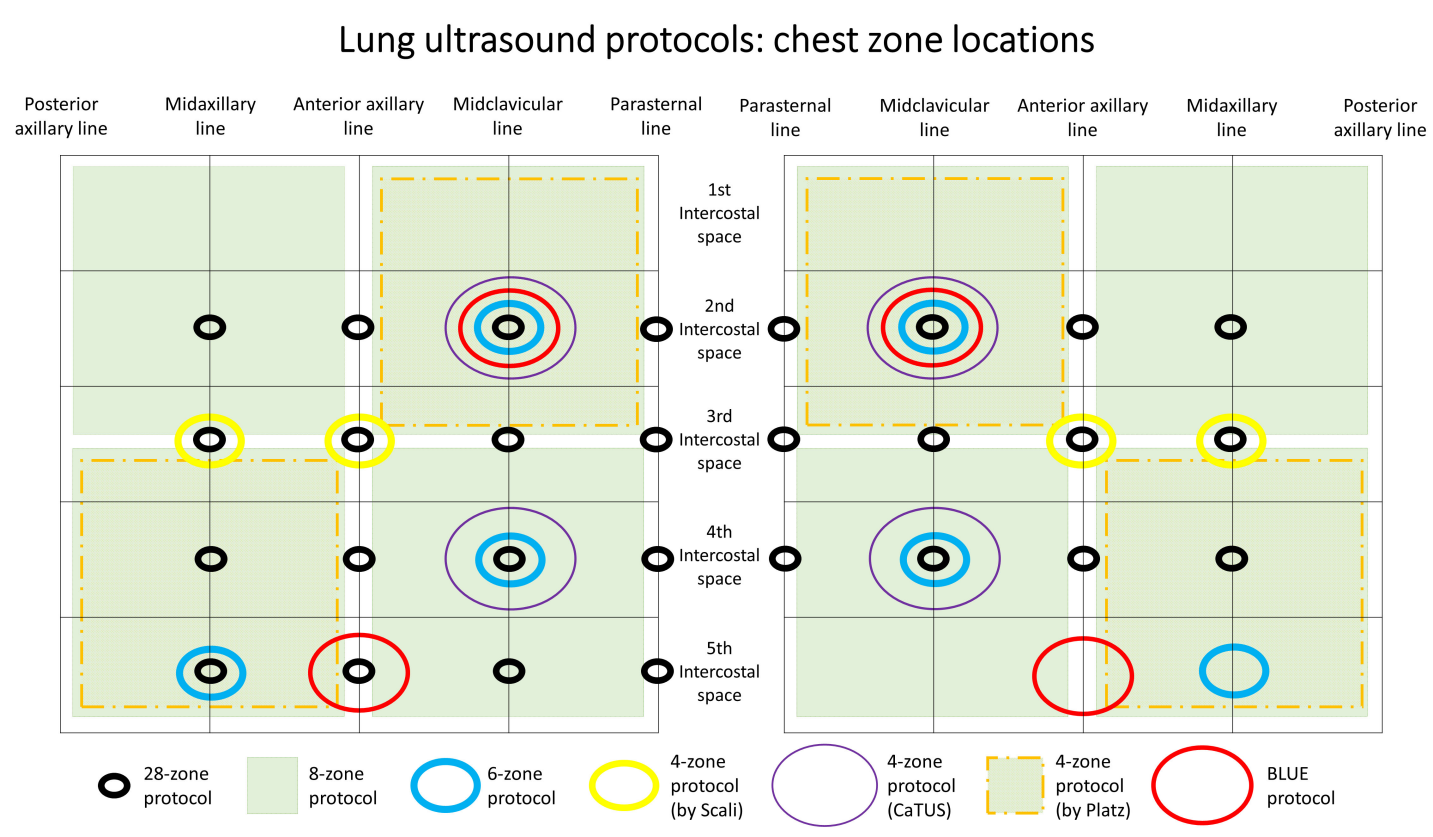
**Chest zone locations with different lung ultrasound methodologies**.

Several imaging protocols have been described in the literature according to different clinical settings, varying from simple 4-zone to 28-zone LUS. The 2012 International evidence-based recommendations on LUS recommended performing LUS with either 8 or 28 chest zones [5]. Recently, an Expert Consensus Document on LUS suggested that at least 6 zones should be examined in HF patients [42]. The enclosed Table 1 (Ref. [11, 12, 13, 16, 20, 21, 22, 23, 25, 26, 29, 30, 31, 47, 48, 49, 50, 51, 52, 53, 54, 55, 56, 57, 58, 59, 60, 61]) describes specific details regarding LUS scanning protocols. Fig. 1 shows chest zone locations with different lung ultrasound methodologies.

**Table 1.** **Description of lung ultrasound techniques (Ref. [11, 12, 13, 16, 20, 21, 22, 23, 25, 26, 29, 30, 31, 47, 48, 49, 50, 51, 52, 53, 54, 55, 56, 57, 58, 59, 60, 61])**.

Lung ultrasound technique – description			^†^Weight of evidence in different settings (from + to +++)		
Scanning protocol	Location of chest zones	- B-line quantification/positive LUS definition	Diagnostic value	Prognostic value	LUS-guided therapy
28 chest zones [16]	28 points: from the 2nd to 4th (5th on the right side) intercostal spaces at the parasternal, midclavicular, anterior axillary, and midaxillary lines	- B-line count [16, 21, 22, 26, 47, 48]	**++**	**+++**	
		- Picano’s congestion grading: mild (6–15 B-lines), moderate (16–30 B-lines), severe: >30 B-lines [11]	- Decompensated status in CHF: B-line count ≥15 (sensitivity = 85%, specificity = 83%; E/e’ ≥15 and/or NT-proBNP >1000 pg/mL as reference)	-AHF (admission)	
		B-line score (positive if ≥1 positive zone bilaterally)		B-line count ≥45 was associated with worse outcomes (death or HHF) at 3–14 months (adjusted HRs from 1.90 to 9.2)	
				-AHF (discharge)	
				B-line count ≥15 was associated with worse outcomes (death or HHF) at 3–6 months (adjusted HRs from 2.5 to 11.7)	
11 chest zones [49]	3 anterior zones (from the sternum to the anterior axillary line, (upper, medium, and lower halves from clavicle to diaphragm) and 3 lateral zones (from the anterior to the posterior axillary line, same subdivision of the anterior area) on right the side; 2 anterior zones (lower zone not assessed due to cardiac interposition) and 3 lateral zones on the left side	- B-line score (sum of positive zone [49]	**+**		
			- Useful in monitoring decongestion; significant correlations with clinical/radiologic congestion score and natriuretic peptides		
8 chest zones [50]	2 anterior zones (from the sternum to the anterior axillary line, subdivided into upper and lower halves from clavicle to the second-third intercostal spaces and from the third space to diaphragm), and 2 lateral zones per side (from the anterior to the posterior axillary line, subdivided into upper and basal halves)	- B-line count [22, 25, 29, 30, 31, 51]	**+++**	**+++**	**+++**
		- B-line score [22, 52, 53, 54, 55, 56, 57]	AHF diagnosis:	CHF setting	LUS-guided therapy (intervention if B-line ≥3) was associated with better outcomes (death, HHF, and urgent HF visits) at 6 months (HR ∼0.55, *p *< 0.05)
		- AHF: positive scan if ≥1 positive zone bilaterally	Sensitivity 65–96%	Positive scanning was associated with adverse outcome (death or HHF) at 6–12 months (adjusted HRs from 1.8 to 4.1)	
		- CHF: positive scan if B-line count ≥3	Specificity 69–96%	AHF (discharge)	
			NPV 88–94%	B-line count ≥5 was associated with worse outcomes (death, HHF, and urgent HF visits) at 6 months (HR 2.63, *p* = 0.033)	
			PPV 91–95%		
6 chest zones [58]	2 anterior zones (2nd and 4th intercostal spaces on the hemiclavicular line), and 1 lateral zone (5th intercostal space on the medium axillary line) per side. 2 additional basal zones on the posterior axillary line for pleural effusion assessment	- B-line score [17, 58]	**++**		
			AHF diagnosis:		
			Sensitivity 91–94%		
			Specificity 84–93%		
			NPV 91–92%		
			PPV 88–92%		
5 chest zones [59]	The surface projections of the 5 major lung lobes	- B-line count (positive scan if B-lines ≥3, CHF setting) [59]		**+**	
				A positive scan independently predicted death of HHF at a median FU of 530 days (adjusted HR 2.9, *p* = 0.011)	
4 chest zones (A) [60]	Four “wet spot” located on the third intercostal space along the midaxillary and anterior axillary lines on both hemithoraces, bilaterally)	- B-line count	**+**	**++**	
		B-line grading congestion during stress ${\mathrm{echocardiography}}^{*}$: absent (0–1 B-line), mild (2–4 B-lines), moderate (5–9 B-lines), and severe (≥10 B-lines) [61]	- Accuracy in detecting B-lines during ${\mathrm{ESE}}^{§}$:	-Severe congestion during SE independently predicted death or nonfatal MI at a median FU of 15 months in a mixed cohort (adjusted HR 3.54, *p* = 0.006)	
			Sensitivity 94%		
			Specificity 100%		
			NPV 88%		
			PPV 100%		
Scanning protocol	Location of chest zones	- B-line quantification/positive LUS definition	Diagnostic value	Prognostic value	LUS-guided therapy
4 chest zones (B) [23]	Apical and mammillary regions on the midclavicular line bilaterally (as part of the CaTUS protocol, also including IVC, E/e’, and pleural effusion assessment)	- B-line score (≥1 positive zone bilaterally) [12, 23]	**+**	**+**	
			- AHF diagnosis (LUS alone):	-Resolution of pulmonary congestion at discharge in AHF (i.e., responder patients) independently predicted mortality at 6 months (HR 0.19, *p* = 0.010).	
			Sensitivity 96%		
			Specificity 81%		
			NPV 88–94%		
			PPV 91–95%		
			- Responders at discharge had larger absolute decrease in E/e’, VAS score and IVCi during treatment and a lower E/e’, VAS score, BNP and IVCi on the day of discharge		
4 chest zones (C) [20]	Upper anterior and basal lateral halves of the 8 chest zone protocol, bilaterally	- B-line count and B-line score [20]	**+**	**++**	
			-Useful in monitoring decongestion in AHF patients	- Admission tertile II (B-lines 5–9) and III (B-lines ≥10) predicted in-hospital adverse outcome (reference tertile I, adjusted HR 2.1 and 4.4, *p* for trend= 0.01	
				- Discharge tertile III (B-lines ≥7) predicted adverse outcome as compared with reference (tertile I, B-lines 0–3) at 3 months (adjusted HR 2.01, *p* = 0.021)	
BLUE Protocol [13]	2 anterior symmetrical regions per lung, an upper BLUE point located at the anterior chest at the midclavicular line on the 2nd-3rd intercostal space, and a lower BLUE point located at the anterior axillary line, just above the nipple	- B-line score (positive if ≥1 positive zone bilaterally) [13]	**+++**		
			- AHF/PO diagnosis:		
			Sensitivity 97%		
			Specificity 95%		
			NPV 99%		
			PPV 87%		

CaTUS, cardiothoracic ultrasound; BLUE, Bedside Lung Ultrasound in Emergency; HR, hazard ratio; ESE, exercise stress echocardiography; SE, stress echocardiography; MI, myocardial infarction; IVCi, inferior vena cava index; VAS, visual analogue scale, PO, pulmonary oedema; HHF, hospitalisation for heart failure, AHF, acute heart failure; CHF, chronic heart failure; NPV, negative predictive value; PPV, positive predictive value. B-line count: sum of B-lines in all zones. B-line score: sum of positive zones (defined as ≥3 B-lines). ^†^The grading system is based on available evidence in the literature (from + to +++). *LUS performed at the end of exercise and beginning of recovery within 1 to 2 min after termination of stress, or extemporaneously at the time of antidote administration in pharmacological stress testing. ^§^28 chest zones as reference.

**Fig. 2.**
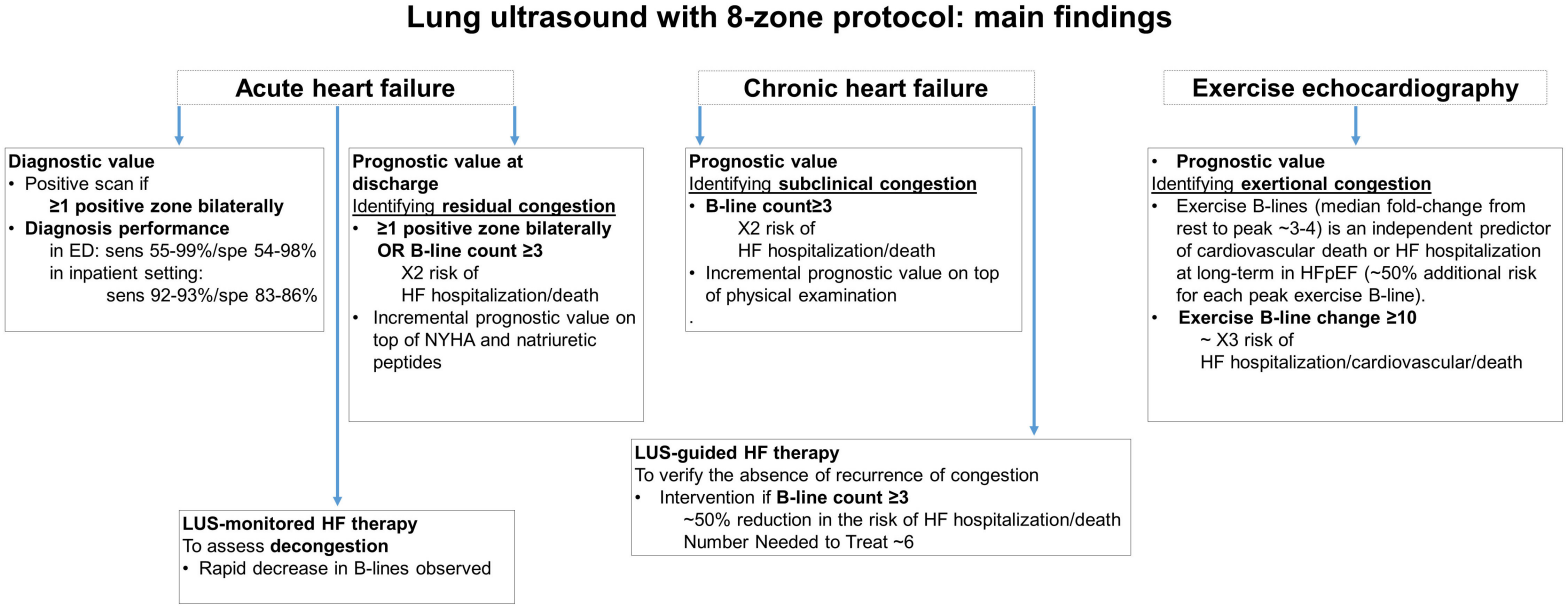
**Different applications and main findings of lung ultrasound methodology with 8-zone protocol**.

**Fig. 3.**
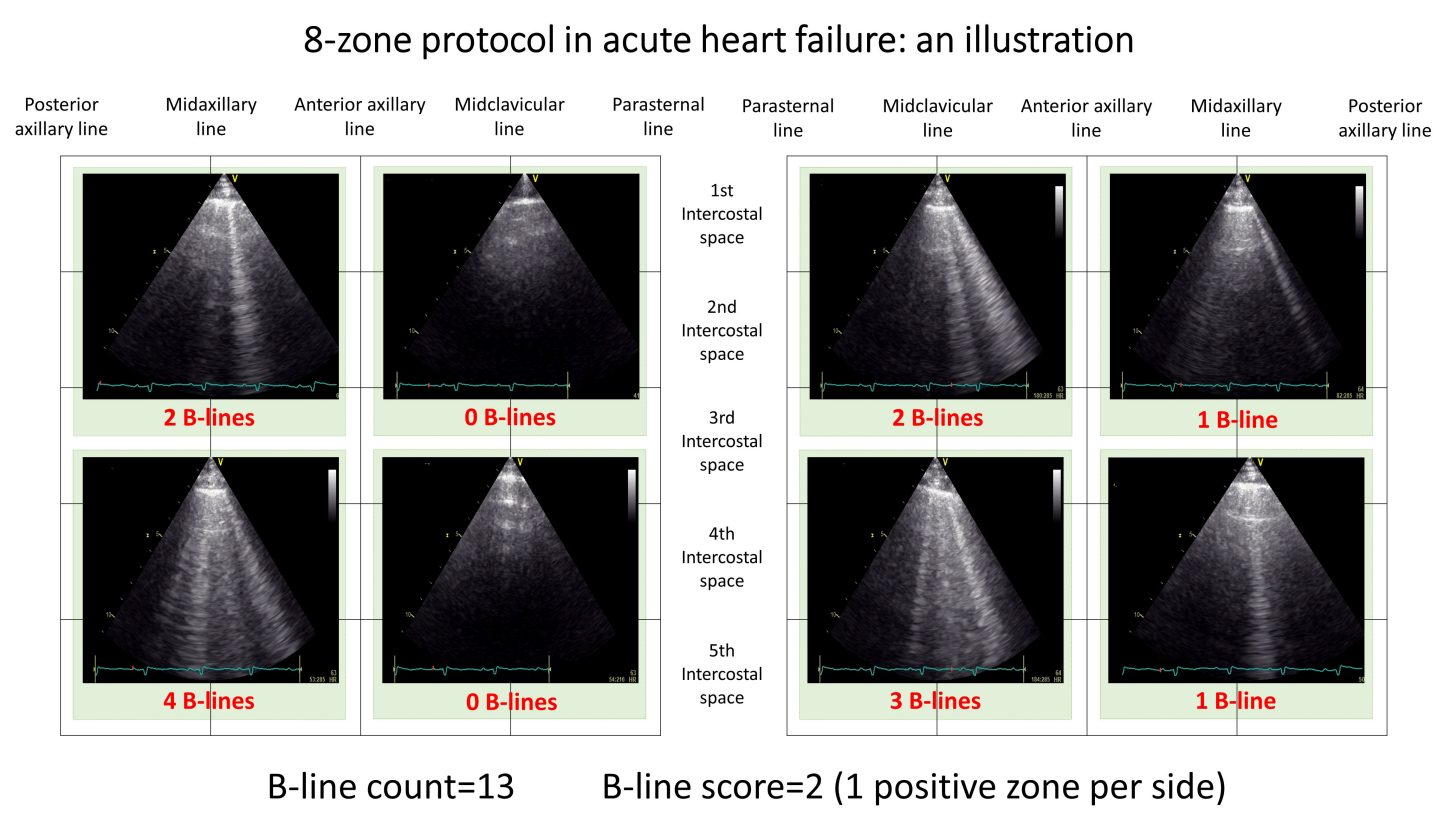
**8-zone protocol application: findings in a patient with acute heart failure**.

The 8-zone protocol, introduced in 2006 by Volpicelli, represents the most versatile scanning method, largely adopted in both acute and chronic settings [25, 50]. Fig. 2 illustrates main findings with 8-zone protocol in different settings, and Fig. 3 shows an illustration in a patient with AHF. The 8-zone protocol consists of 2 anterior zones (from the sternum to the anterior axillary line, subdivided into upper and lower halves from the clavicle to the second-third intercostal spaces and from the third space to the diaphragm), and 2 lateral zones (from the anterior to the posterior axillary line, subdivided into upper and basal halves) per side. Volpicelli proposed another scanning protocol with 11 zones, 6 on the right side and 5 on the left side [49]. The 6 chest zone protocol was derived from the 8-zone protocol by locating six specific points of intersections on the hemiclavicular and midaxillary line [58].

The 28-zone protocol, more time-consuming in comparison with the 8-zone protocol, has had broad use particularly in the chronic ambulatory setting [26, 62] and at discharge from an HF hospitalisation [22]. It includes bilateral scanning from the 2nd to 4th (5th on the right side) intercostal spaces at the parasternal, midclavicular, anterior axillary and midaxillary lines [16, 22].

Three different 4-zone protocols have been reported in the literature. The first protocol (by Scali *et al*. [60]) was derived from the 28-zone protocol, in which four “wet spots” (i.e., the zones with highest B-line density, located on the third intercostal space along the midaxillary and anterior axillary lines on both sides) were identified. Correlation between B-line counts obtained with the 2 scanning protocols was excellent [60]. Another 4-zone protocol is part of the cardiothoracic ultrasound (CaTUS) protocol, including apical and mammillary regions on the midclavicular line bilaterally [23]. A third 4-zone protocol was proposed by Platz *et al*. [20], which includes upper anterior and basal lateral halves of the 8-zone protocol, bilaterally. In critical ICU patients, the bedside lung ultrasound in emergency (BLUE) protocol consists of six zones, 4 of which are investigated for the diagnosis of PO [13]. Finally, a 5-zone protocol was proposed by Gustafsson *et al*. [59].

## 3. Lung Ultrasound in Acute Heart Failure

### 3.1 Diagnostic Value

There are several settings for LUS implementation in patients with dyspnoea and presumed AHF. ED physicians often face the challenge of differentiating between pulmonary and cardiac causes of acute respiratory failure; a timely diagnosis of AHF has major implications since early treatment can improve short-term outcomes [63].

In a prehospital setting, LUS feasibility was deemed excellent, with a very high negative predictive value (NPV) (approximately 95%) in ruling out AHF, and with a good positive predictive value (PPV) (77.3%) [15]. Additionally, LUS (NPV 100%, PPV 96%) was the best single method for diagnosing AHF in the prehospital setting (i.e., performed immediately after arrival of the patient at the ED but prior to any administered treatment) when compared with NT-proBNP and physical examination [64].

In the ED, the sensitivities and specificities of LUS for AHF range from 55 to 100% and from 54 to 98%, respectively [12, 17, 43, 50, 53, 54, 55, 57, 58, 65, 66, 67, 68, 69, 70, 71]. The diagnostic value of LUS (usually using the 8-zone method) has mostly been assessed alone or, in some studies [12, 57, 65, 67, 69, 70], integrated with various cardiac ultrasound protocols, including assessment of inferior vena cava diameter, left ventricular ejection fraction (LVEF), severity of mitral regurgitation, and estimated left ventricular filling pressures by E/e’.

In a large multicenter, prospective cohort study by Pivetta *et al*. [17] (N = 1005), a 6-zone LUS approach had a significantly higher accuracy for AHF (sensitivity 97%, specificity, 97.4%) compared to clinical workup alone, chest X-ray or NT-proBNP, and showed an additive diagnostic value on top of a standard approach [net reclassification improvement (NRI) 19.1%, 95% confidence interval (CI) 14.6–23.6, *p *< 0.01]. In a more recent study, the same group randomised 518 ED patients to 8-zone LUS or chest-X ray/NT-proBNP after an initial clinical work-up. LUS exhibited a higher accuracy for AHF than the chest-X ray/NT-proBNP approach (AUC 0.95 vs. 0.87, *p *<0.01) [53] and reduced diagnostic misclassification resulting from clinical approach alone by 8 cases/100 patients [53].

Recently, Buessler *et al*. [43] compared the diagnostic accuracies of various LUS scanning protocols [i.e., 4- (BLUE protocol), 6-, 8-, and 28-zone protocols] in diagnosing AHF in a cohort of 117 ED patients admitted for acute dyspnoea and diagnostic uncertainty. Among the studied scanning protocols, the 8-zone protocol (≥1 bilateral positive zone) had the highest diagnostic accuracy (C-index 74.0%), while also showing the highest additive diagnostic value on top of a validated clinical score (C-index increase 6.9; 95% CI, 1.6–12.2, *p* = 0.010). Thus, in ED settings, the less time-consuming 8-zone protocol seemingly represents the most reliable and useful LUS method in diagnosing AHF.

In an inpatient setting (cardiology or internal medicine department), various studies have also confirmed the diagnostic accuracy of LUS [16, 56, 72, 73, 74, 75], with sensitivities and specificities ranging from 81 to 97%, and from 79 to 86%, respectively. Of particular interest, the diagnostic accuracy of LUS in detecting high estimated left ventricular pressures (as assessed by E/e’ ≥14) was excellent both in HFpEF (AUC 0.94) and HFrEF (AUC = 0.84) [75].

Overall diagnostic value can also be derived from various meta-analyses published in the last decades, including different patient populations. Al Deeb *et al*. [76] reported that the sensitivity and specificity of B-lines in diagnosing acute cardiogenic pulmonary oedema was 94% and 92%, respectively, in a mixed population (N = 1075) encompassing patients from ICU, internal medicine wards, and prehospital-ED settings with different LUS methods, which also included count-based methods. Martindale *et al*. [77] performed a meta-analysis including 1918 patients (8 studies in an ED setting), assessed only with score-based methods (primarily with the 8-zone protocol), with a reported sensitivity and specificity of 85% and 93%, respectively. A more recent meta-analysis by McGivery *et al*. [78] (1861 ED patients with undifferentiated dyspnoea; 7 studies adopting the Volpicelli method), reported comparable sensitivity (83%), albeit with lower specificity compared with the study of Martindale *et al*. [77]. In a meta-analysis including a mixed population of 1827 patients from ED or internal medicine settings, Maw *et al*. [79] compared the accuracy of LUS with chest-X-ray in which LUS was found to be more sensitive than chest-X ray in diagnosing AHF (88% vs. 73%, relative sensitivity ratio = 1.2, *p *< 0.001), with no differences observed in terms of specificity (90% for both methods).

In addition to B-lines, pleural effusion (i.e., an anechoic or hypoechoic space between the two pleural layers) also has diagnostic value for AHF [53, 58, 77]. Overall, unilateral (mostly on the right side) or bilateral pleural effusions are encountered in about 60% of patients with AHF [53] and are associated with a higher risk of death or HF hospitalisation when combined with B-lines in outpatients [59]. However, the identification of pleural effusion by LUS only has moderate accuracy for the diagnosis of AHF (sensitivity ~60%, specificity ~70%) [77]. As compared to pleural effusion alone, the coexistence of B-profile and pleural effusion increases LUS specificity, but may decrease sensitivity given that not all patients with acute cardiogenic dyspnoea show pleural effusion [58]. Finally, large pleural effusions may interfere with B-line quantification as they can induce B-lines that are not related to pulmonary congestion (but rather to passive changes in lung tissue compressed by pleural effusion).

### 3.2 Monitoring Decongestion

The potential utility of LUS in monitoring pulmonary decongestion in response to AHF treatment has been assessed in several studies [20, 23, 24, 47, 49, 80, 81]. In a mixed cohort of 340 patients admitted for dyspnoea, Frassi *et al*. [81] showed that in the subgroup (N = 70) exhibiting a clinical response to treatment (i.e., decrease in NYHA functional class ≥1), the B-line count (assessed with the 28-zone protocol) decreased prior to discharge (42 ± 32 vs. 15 ± 18, *p *< 0.0001), 6 ± 4 days after the initial assessment on admission. Similar B-line count changes were also reported in (a) a cohort of 100 patients admitted for AHF with LUS performed on admission and at discharge (48 ± 48 vs. 20 ± 23, *p *< 0.0001) [47], and (b) a small cohort of 25 ED patients after 24 hours of i.v. diuretic therapy (53 ± 17 vs. 32 ± 14, *p *< 0.001) [80]. Volpicelli *et al*. [49] furthermore showed a significantly decreased pattern in median B-line score (assessed with an 11-zone protocol) after a mean period of 4.2 ± 1.7 days of medical treatment in a cohort of 70 AHF patients [8 (IQR 3–9) vs. 0 (IQR 0–7), *p *< 0.0001]. A more rapid B-line clearance was also demonstrated with the 11-zone protocol in a cohort of 41 ED patients admitted for PO, in which the mean B-line score (0–2 for each zone, 1 for ≥3B-lines, 2 for white lung) decreased by 54% after 3 hours of medical treatment (from 1.59 ± 0.40 to 0.73 ± 0.44, *p *< 0.001), with an additional 47% reduction from 3 to 24 hours (from 0.73 ± 0.44 to 0.38 ± 0.33, *p *< 0.001). Overall, B-line clearance during pulmonary decongestion was consensual and significantly correlated with improvement in dyspnoea and physical examination findings [20, 23, 24, 49, 80], as well as with the radiologic congestion score index [49].

Of note, data on the concomitant decline of B-line counts and natriuretic peptides are conflicting [23, 49, 80]. Natriuretic peptide clearance in an acute setting appears to exhibit slower kinetics when compared with B-line variations to therapy, especially in the presence of renal failure [23]. Divergent results have also been reported for E/e’ [23, 80], although definitive conclusions cannot be drawn given the small size of these studies. The most probable underlying reasons for these discrepancies are the distinct kinetics of the various, congestion variables, as well as the fact that each congestion variable measures a different area of congestion, e.g., LUS specifically investigates pulmonary congestion whereas E/e’ investigates intracardiac pressure, which can induce pulmonary congestion differently depending on the physical properties of the alveolocapillary border and the level of lung inflammation.

### 3.3 Prognostic Value

Pulmonary congestion assessed by LUS has been shown to be associated with adverse outcome in patients hospitalised for AHF, regardless of the timing of quantification during the hospital stay [82]. Indeed, its prognostic value has been demonstrated at admission [19, 20, 21, 47, 82, 83] and at discharge [20, 22, 47, 48, 52, 82, 83, 84]. The enclosed Table 2 (Ref. [20, 21, 22, 25, 29, 47, 48, 51, 62, 82, 84, 85]) describes specific details regarding prognostic value of LUS in different settings.

**Table 2.** **Description of studies included in the review to assess prognostic value of LUS (Ref. [20, 21, 22, 25, 29, 47, 48, 51, 62, 82, 84, 85])**.

Author year	Population	Total	${\mathrm{FU}}^{\wedge }$	Chest zones; position	Age	LVEF%	B-lines quantification (stratification level)	Total events	Event details	HR/RR (CI) *p* value ^a^	Covariates of adjustment
Outpatient setting											
Curbelo *et al*., 2018 [51]	CHF	99	12 m	8; NA	84.2 (6.5)	57.5 (14.9)	Stratification ≥3; ≥5; ≥10; ≥15	36	Death: 12	Multi: 1.8 (0.8–4.0) *p* = NA	Gender, age, LVEF, NYHA class, GFR, Hb
									HHF: 35	Uni: 1.7 (0.8–3.5)	
Dwyer *et al*., 2018 [29]	Ambulatory HF patients and hypertensive patients	119 HF (total = 230)	12 m	8; supine	66 (20–93)	55 (46–62)	Stratified ≥3 B-lines	28 (patients with HF)	Death: 10	Multi: 2.62 (1.15–5.96) *p* = 0.022	Age, sex
									HHF: 21	Uni: 2.46 (1.15, 5.26) *p* = 0.020	
Pellicori *et al*., 2019 [62]	CHF	342	7.8 (4.5–11.7) m	28; near-supine position	75 (68–82)	45 (14)	Continuous and stratified 0–3; 4–13; ≥14	60	Death: 25	Multi: 1.54 (0.68–3.48), *p* = 0.29	Age, NYHA class (III vs. I/II), urea, Hb and log NTproBNP + JVD ratio
									HHF: 35	Uni: 4.33 (2.09–8.98), *p *< 0.001	
Platz *et al*., 2016 [25]	Patients with HF (NYHA II-IV)	195	6 m	8; NA	66 (24–93)	34 (23–51)	Stratified Tertiles	50	Death: 15	Multi: 4.08 (1.95–8.54), *p *< 0.001	Age, gender, NYHA class III or IV, and congestion score
							(0; 1–2; ≥3)		HHF: 48		
									Urgent visit: 9	Uni: 3.78 (1.88–7.63), *p *< 0.001	
Domingo *et al*., 2021 [85]^$^	At least 1 HHF and/or LVEF <40%	577	31 ± 7.1 m	8; semi-supine	68.8 (12.3)	45.4 (12.6)	Continuous & quartiles	157	Death: 111	Multi: 1.26 (0.89–1.77), *p* = 0.19	Age, sex, BMI, ischemic aetiology, Log HF duration, NYHA, LVEF, diabetes, hypertension AF/AFlu, renal insufficiency, ACEI-ARB, BB, Loop diuretics, Hydralazine, nitrates
							(Q1 = 1 B-lines,				
							Q2 = 3 B-lines,				
							Q3 = 7 B-lines, Q4 ≥ 8)		HHF: 74	Uni: 1.66 (1.20–2.30), *p* = 0.002	
AHF (admission)											
Gargani *et al*., 2015 [47]	Dyspnoea and/or suspicion of AHF	100	6 m	28; supine or near supine	70 (11)	37 (14)	Stratified >50	14	Death: 4	Multi: 4.87 (0.88–27.06), *p* = 0.07	NYHA class, Hb, NT-proBNP at discharge, BL at admission and discharge
			(5.3 ± 1.6)						HHF: 14	Uni: 5.83 (1.82–18.62), *p* = 0.002	
Coiro *et al*., 2016 [82]	ADHF	50 (total 110)	3 m	28; supine or near supine	73 (2)	37 (2)	Stratified ≥45	33 in whole cohort	Death: 16	Multi: 9.20 (1.82–46.61), *p* = 0.007	B-lines categorical, NYHA class, IVC diameter, echocardiographic (E/A >2) creatinine clearance
									HHF: 26	Uni: 5.85 (1.86–18.46), *p* = 0.003	
Gargani *et al*., 2021 [21]^$^	HFrEF	296 HF (total = 1021)	14.4 m	28; supine	70 (62–76)	30 (23–35)	Stratified >30; 45	82	Death: 40	Multi: 1.90 (1.14–3.16)	Age, creatinine, NYHA class
	HFpEF				73 (66–80)	55 (50–60)			HHF: 42		
AHF (discharge)											
Coiro *et al*., 2015, 2016 [22, 82]	ADHF	60	3 m	8 and 28; supine or near supine	72.1 (10.2)	47.5 (27.2–52)	Continuous and stratified (≥15; ≥30)	18	Death: 10	Multi: 4.1 (0.95–14.63), *p* = 0.055	Clinical (lung rales, ankle oedema, NYHA class ≥III), biological
											(MDRD) creatinine clearance <50 mL/min, and echocardiographic
									HHF: 15	Uni: 5.8 (2.1–16.3), *p* = 0.001	(E/A >2+DT <150 ms, and IVC diameter)
Gargani *et al*., 2015 [47]	Dyspnea and/or suspicion of AHF	100	6 m	28; supine or near supine	70 (11)	37 (14)	Stratified >15 and ≤15	14	Death: 4	Multi: 11.74 (1.3–106.16), *p* = 0.028	NYHA class, Hb, NT-proBNP at discharge, BL admission & discharge
									HHF: 14	Uni: 24.12 (3.15–184.55), *p* = 0.002	
Rivas-Lasarte *et al*., 2020 [84]	ADHF without sub-clinical congestion (n = 59)	100 (total 123)	6 m	8; semi-recumbent	65 (14)	38 (13)	Stratified B-lines (≥5 B-lines)	28	Death: 3	Multi: 2.63 (1.08–6.41), *p* = 0.033	Age, renal insufficiency, AF, anaemia, NYHA class, NT-proBNP levels, systemic clinical congestion, group intervention (LUS-guided strategy)
	ADHF with sub-clinical congestion (n = 41)				70 (10)	39 (14)			HHF: 19	Uni: NA	
									Urgent visit: 12		
Platz *et al*., 2019 [20]	Acute HF	132 (349)	3 m	4; semi-recumbent	72 (60–83)	41 (17)	Stratified B-lines (3 tertiles: 0–3; 4–6; ≥7)	42	Death: 13	Multi: 1.45 (0.6–3.46), *p* = 0.41	Age, log creatinine, SBP (stratified by sex)
					76 (64–86)	39 (14)			HHF: 32	Uni: 1.42 (0.61–3.32), *p* = 0.42	
Rueda-Camino *et al*., 2021 [48]	ADHF with preserved LVEF	103	3 m (2.4 ± 0.9)	28	82.2 (9.1)	61.1 (7.0)	Stratified >15 and ≤15	28	Death: 1	Multi: 2.46 (1.11–5.46), *p* = 0.03	Sex, functional class, Charlson’s comorbidity index, Barthel’s index, respiratory comorbidity (COPD, asthma, and sleep apnoea-hypopnea syndrome)
									HHF: 27	Uni: 2.39 (1.12−5.12), *p* = 0.024	

AA, aldosterone antagonist; ACEI, angiotensin converting enzyme inhibitor; AF, atrial fibrillation; AFlu, atrial flutter; ARBs, angiotensin receptor blockers; ARNI, angiotensin receptor neprilysin inhibitor; ADHF, acute decompensated heart failure; AHF, acute heart failure; CAD, coronary artery disease; CCB, calcium channel blocker; CHF, congestive heart failure; COPD, chronic obstructive pulmonary disease; CRT, cardiac resynchronisation therapy; CV, cardiovascular; FU: follow-up; Hb, haemoglobin; HHF, hospitalization for HF; HR, hazard ratio; GFR, glomerular filtration rate; ICD, implantable cardioverter defibrillator; ILD, interstitial lung disease; IVCd: inferior vena cava diameter; LVEF, left ventricular ejection fraction; LUS, lung ultrasound; MRA, mineralocorticoid antagonist; multi, multivariable; NA: not available; NYHA, New York Heart Association; RR, risk ratio; SBP, systolic blood pressure; Uni, univariable; VAD, ventricular assist device. *B-lines >15 in 28 zones-LUS or ≥3 (or ≥5) in 8 zone-LUS in outpatients and discharge patients studies and B-lines ≥45 (or ≥50) in admission LUS studies. ^FU mean or median duration reported in parenthesis. a adjusted hazard ratio presented for selected cut-offs. $ HRs provided by the authors. Hazard ratios are presented for combined end-points.

In a study by Platz *et al*. [20] using the 4-zone protocol, a B-line count ≥10 on admission (B-lines 0–4 as reference) independently predicted the composite outcome of death, ICU admission or cardiac arrest, need for left ventricular assist devices or inotropes in a cohort of 349 AHF patients [adjusted odds ratio (OR) 4.43; 95% CI 1.43–13.67; *p* = 0.010]. Increased B-line count upon admission (8-zone protocol) was found associated with adverse outcome (composite of death or HF hospitalisation) at 60 days after discharge in a cohort of 216 patients with new-onset or worsening HF, but only in non-obese patients [i.e., body mass index (BMI) <30 kg/$m^{2}$] [19]. Of note, the authors suggested that obesity/elevated BMI should be considered not only when interpreting natriuretic peptides but also B-line count for several possible mechanisms including differing treatment response, lower degree of pulmonary congestion and B-line specificity due to a higher prevalence of pulmonary fibrosis or chest infection [19]. With regard to the prognostic value of specific B-line count cut-offs, our group found that a B-line count ≥45 (assessed with the 28-zone protocol within 3 days after admission) was significantly associated with a higher risk of death or HF hospitalisation at 90 days [hazard ratio (HR) 4.60; 95% CI 1.73–12.25; *p* = 0.002], independently of atrial fibrillation status and LVEF [82]. Using the same scanning protocol, an admission B-line count >30 significantly predicted the composite outcome of cardiac death or HF hospitalisation at long-term only in the HFpEF subgroup in a cohort of 296 AHF (199 with HFrEF, 97 with HFpEF) by multivariable analyses (HR 5.54, 95% CI 1.35–22.73, *p* = 0.017) [21].

Residual pulmonary congestion as assessed by B-line count at time of discharge has been shown to identify a subset of patients with AHF at high risk of readmission or death [20, 22, 47, 48, 52, 82, 83, 84], while its prognostic value appeared to be far superior to admission B-lines when assessed in the same study [47, 83].

In 2015, two different research groups first demonstrated the prognostic value of discharge B-lines as assessed with the 28-zone protocol [22, 47]. Gargani *et al*. [47] showed that a B-line count >15 was independently associated with a higher risk of HF hospitalisation at 6 months in a cohort of 100 patients (HR 11.74, 95% CI 1.30–106.16; *p* = 0.028). Similarly, in a study by Coiro *et al*. [22] B-lines ≥30 significantly predicted the combined endpoint of death or HF hospitalisation at 3 months (HR 5.66, 95% CI 1.74–18.39, *p* = 0.04). Similar results were reported with the scanning method of Volpicelli *et al*. [49] derived by grouping the 28 scanning sites in the 8 corresponding zones. Both 1 and 2 positive zones per side were also significant predictors of the combined endpoint by multivariable analysis [22]. Additionally, B -lines enabled significant patient risk reclassification (continuous NRI 65%, *p* = 0.03) on top of usual risk stratification (i.e., NYHA and BNP), thus suggesting a relevant improvement in risk assessment at discharge following HF hospitalisation [22]. Comparable results were also reported in another cohort of 100 AHF patients with the 8- zone protocol, using both B-line score and count, whereby 1 or 2 positive zones per side and B-lines >15 or >30 (as assessed with the 28-zone protocol) were all significantly associated with the combined endpoint at 100 days (HR >2 and *p *< 0.03 for all) [52]. In another study using the same scanning protocol, B-line count also predicted the combined endpoint of death or HF hospitalisation at 6 months (HR per each B-line increase 1.16, 95% CI 1.11–1.21, *p *< 0.001), for both HFrEF and HFpEF [83]. In a cohort of 132 AHF patients, a clear stepwise association of B-line tertiles (assessed by the 4-zone protocol by Platz *et al*. [20]) with an increased risk of death or HF hospitalisation at 180 days was observed. This latter relationship was time-varying (i.e., stronger closer to discharge), and persisted after adjusting for major clinical variables, including NT-proBNP [20]. Similar results were also found for both 4- and 8-zone LUS in the subset of 123 patients with both scanning protocols [20]. Persistent pulmonary congestion at discharge (defined as B-lines ≥15 with the 4-zone protocol by Scali *et al*. [60]) in addition to chronic kidney disease (CKD, estimated glomerular filtration rate <60 mL/min/1.73 $m^{2}$) identified a subgroup at high risk for death or HF hospitalisation at 12 months [86]. Additionally, the combination of clinical congestion and CKD was associated with increased levels of TNF-alpha, which in turn attenuated the direct relationship between the two risk markers and outcome [86]. In another cohort of 170 outpatients with suspected new-onset HF, B-lines were moderately correlated with other markers of inflammation (i.e., growth differentiation factor 15, IL-6, and high sensitivity C-reactive protein) [87]. Taken together, these data suggest that inflammation may have a pivotal role in the links between congestion, renal dysfunction and adverse outcome.

The presence of subclinical pulmonary congestion assessed with the 8-zone protocol at discharge (i.e., “dry lung” on auscultation with a B-line count ≥5) was associated with a higher risk of urgent visit, hospitalisation for worsening HF and death at 6 months comparatively to those without congestion in a cohort of 123 AHF patients. This risk was notably similar to those discharged with rales (HR ~2.7 for both) [84].

Finally, the overall prognostic value of both admission and discharge B-lines has been assessed in several meta-analyses [88, 89, 90]. A fixed-effect meta-analysis by Rastogi *et al*. [90] which included studies published from 2010 and 2021, yielded the following cut-off points for pooling risk estimates: (i) admission: B-lines ≥45 for 28 chest zones, and (ii) discharge: B-lines ≥15 in 28 zones (~0.5 B-line/zone); B-lines ≥3 in 5 to 8 zones (~0.4 B-line/zone); B-lines ≥4 in 4 zones (1 B-line/zone). A higher number of B-lines during an AHF hospitalisation was associated with an increased risk of primary outcome after adjusting for clinically relevant variables, irrespective of the timing of assessment [relative risk (RR) at admission 2.32, 95% CI 1.46–3.70, *p* = 0.0004, $I^{2}$ = 50.92%; RR at discharge 2.46, 95% CI 1.56–3.86; *p* = 0.0001, $I^{2}$ = 0.00%].

## 4. Lung Ultrasound in Chronic Heart Failure

### 4.1 Assessment of Pulmonary Congestion by Lung Ultrasound and Its Association with Other Established Tools

Various studies have highlighted the usefulness of LUS in identifying a decompensated HF status in an outpatient setting. In a cohort of 97 HFrEF outpatients, Miglioranza *et al*. [26] found that LUS (assessed with the 28-zone protocol) yielded a C-statistic of 0.89 in identifying a decompensated status (NT-proBNP >1000 pg/mL and/or E/e’ ≥15 as reference) and provided the best accuracy for a cut-off of 15 B-lines (sensitivity 85%, specificity 83%). Similar results were reported when taking a more comprehensive multi-parametric approach as reference, including clinical score, chest X-ray, and the 6-minute walk test [26]. Of note, all patients with a B-line count ≥15 presented a pattern of multiple bilateral B-lines, while B-line count was well correlated (r = 0.7) with NTproBNP and E/e’.

### 4.2 Prognostic Value

Pulmonary congestion assessed by LUS is associated with worse prognosis in ambulatory patients [25, 27, 28, 29, 51, 62, 85, 91] (see Table 2 for more details). Platz *et al*. [25] first demonstrated the prognostic value of B-lines (assessed with the 28-zone protocol) in a cohort of 195 NYHA class II–IV HF outpatients. Patients in the third tertile (B-line count ≥3) had a four-fold higher risk of death or HF hospitalisation at 6 months (adjusted HR 4.08, 95% CI 1.95–8.54, *p *< 0.001) compared with those in the first tertile. In addition, LUS provided incremental prognostic value when compared with both lung auscultation and a clinical congestion score (including crackles, jugular venous distension, lower extremity oedema). Similarly, other cohorts further validated that LUS findings were associated with a higher risk of death or HF hospitalisation in an ambulatory setting (details in Table 2) [ 27, 29, 51]. This prognostic value was confirmed in two selected cohorts of HFrEF and HFpEF patients [28, 91]. In addition, in HFrEF outpatients, a B-line count ≥30 assessed with the 28-zone protocol was found to be the strongest predictor of PO admission at 120 days (HR 8.62; 95% CI: 1.8–40.1; *p* = 0.006) when compared with other established clinical, laboratory and radiologic prognosticators [91]. In HFpEF patients, both B-line count (assessed with the 28-zone protocol) and NTproBNP exhibited similar accuracy (AUC ~0.86 for both parameters) in predicting the primary outcome, consisting of a composite of hospitalisation for worsening HF, loop diuretic dose escalation and death, at a mean follow-up of 26 months (N = 97) [28], while a B-line count >15 significantly increased the likelihood of adverse outcome with an adjusted HR of 15.47 (*p* = 0.01). The overall prognostic value of LUS in HF outpatients has also been assessed in different meta-analyses [89, 90]. In a recent fixed-effect meta-analysis including 5 studies and 1332 HF outpatients, the following cut-off points for pooling the risk estimates were used: ≥15 using the 28-zone protocol (~0.5 B-line/zone), ≥3 using the 5- to 8-zone scanning protocols [90]. B-line count was associated with an increased risk of primary outcome, irrespective of the setting (outpatient clinic RR: 1.66, 95% CI 1.28–2.15, *p* = 0.0001, $I^{2}$ = 57.5%). However, as with other meta-analyses [89], these results should be interpreted with caution in the context of a heterogeneity observed in these studies, possibly due to the use of different LUS protocols/B-line thresholds, statistical adjustment and HF quality of care.

### 4.3 LUS-Guided Therapy

The usefulness and prognostic impact of LUS in addition to standard care in the management of HF outpatients has recently been assessed in several studies (Table 3, Ref. [30, 31, 92]). Rivas Lasarte *et al*. [30] randomised 123 outpatients discharged from AHF to either standard follow-up (N = 62) or a LUS-guided follow-up (N = 61). In both groups, patients were treated according to current guidelines, and followed the same schedule of visits after hospital discharge. In the LUS-group (assessed with the 8-zone protocol), treating physicians were encouraged to modulate diuretic therapy in accordance with the recorded B-lines during follow-up; a B-line count ≥3 was considered to indicate pulmonary congestion. LUS-guided treatment was associated with a significantly lower risk of urgent visits, hospitalisation for worsening HF and death from any cause when compared with standard follow-up (HR 0.52, 95% CI 0.27–0.99, *p* = 0.049), with a number needed to treat (NNT) = 5. Differences in the primary endpoint were primarily attributable to an increased number of urgent visits for worsening HF. These findings were confirmed in another trial which randomised 126 HF outpatients discharged from AHF hospitalisation, with similar scanning protocol, pulmonary congestive status criteria according to LUS findings, composite endpoint and study intervention [31]. Patients in the LUS-guided treatment group had better outcomes compared with standard follow-up at 6 months (HR 0.55, 95% CI 0.31–0.98, *p* = 0.044; NNT = 6), mainly driven by a reduction in urgent HF visits. Marini *et al*. [92] further corroborated the added value of a LUS-guided treatment in a larger trial of 244 stable HFrEF (LVEF <45%) outpatients: patients in the LUS group exhibited a lower risk of hospitalisation for AHF at 90 days (relative risk =0.44, 95% CI 0.23–0.84; *p* = 0.01; NNT = 8.4). Overall, the LUS strategy was associated with a significant decrease in natriuretic peptide during the follow-up period [30, 31] as well as a slightly increased mean furosemide dose during the study period [30, 31]. No differences were found in terms of adverse events (i.e., acute kidney injury, hypokalaemia, hypotension), or evidence-based HF treatment. Two recent meta-analyses assessed the overall impact of LUS-guided HF therapy derived from the above 3 studies [90, 93]. With regard to HF hospitalisation, Mhanna *et al*. [93] reported no significant differences in the rates of HF hospitalisation between the two groups (RR 0.65; 95% CI 0.34–1.22; *p* = 0.18; $I^{2}$ = 49%). As expected, LUS-guided therapy was associated with a significantly lower rate of urgent HF visits (RR 0.32; 95% CI 0.18–059, *p* = 0.0002; $I^{2}$ = 49%). Rastogi *et al*. [90] reported pooled estimates for HF hospitalisation as well as the combined outcome (urgent visits for worsening of HF, hospitalisation for HF and mortality), with a significant difference in favour of a LUS-guided HF therapy for both endpoints [(RR 0.50, 95% CI 0.35–0.72, *p* = 0.001, $I^{2}$ = 0.00%) and (RR 0.62, 95% CI 0.40–0.87, *p* = 0.007, $I^{2}$ = 41.01%)], respectively. Differences in terms of statistical methodology (random- vs. fixed-effect meta-analyses) may have contributed to moderately different results with regard to HF hospitalisation. Altogether, a LUS-guided strategy for HF therapy has demonstrated its usefulness in improving both short- and mid-term prognosis of HF patients, being able to significantly reduce HF hospitalisation or urgent visit.

**Table 3.** **Clinical trials comparing LUS-guided treatment in comparison to standard treatment in HF patients (Ref. [30, 31, 92])**.

Author year	Study design	Population description	Chest zones; position	B-lines quantification	FU	Total N	Number in each group	Age	LVEF%	Total events	Deaths	HHF	Urgent visits	HR/RR (CI) *p* value	Covariates of adjustment	Main exclusion criteria
Rivas-Lasarte *et al*., 2019 [30]	Single-blind randomized clinical trial	HF patients (HF defined by shortness of breath, pulmonary congestion on X-ray and high NT-proBNP values in the first 24 h of admission	8; semi-recumbent	Counts	6 m	123	control group (n = 62)	69 (11)	39 (15)	25	2	13	13	0.52 (0.27–0.99), *p* = 0.049	NA	Inability to attend FU visits; life expectancy of <6 months, haemodialysis; presence of severe lung disease preventing LUS interpretation
							LUS group (n = 61)	69 (13)	39 (14)	14	3	14	3			
Araiza-Garaygordobil *et al*., 2020 [31]	Single-blinded, randomized controlled trial	ADHF patients	8; semi-recumbent	Counts	6 m	126	control group (n = 63)	63 (51–73)	34.9 (14)	30	6	8	25	0.55 (0.30–0.99), *p* = 0.044	Adjusted for sex, age, and NT-proBNP >2322 pg/mL	Severe lung disease preventing LUS interpretation, lack of will to participate, life expectancy shorter than 6 months, chronic kidney injury with Egfr <15 mL/min/1.73 $m^{2}$, death during index hospitalization or a surgically correctable cause of HF
							LUS group (n = 63)	62 (52–71)	30 (15.2)	20	9	4	9			
Marini *et al*., 2020 [92]	Randomised multi-centre non-blinded study	Chronic HF and optimised medical therapy with LVEF <45%	8; NA	NA	3 m	244	control group (n = 117)	69.79 (11.34)	30.73 (8.43)	25	4	25	NA	0.44 (0.23–0.84), *p* = 0.01 (RR for HHF)	NA	NA
							LUS group (n = 127)	73.22 (10.94)	32.16 ± 9.64	12	5	12	NA			

ACEI, angiotensin converting enzyme inhibitor; ARBs, angiotensin receptor blockers; ARNI, angiotensin receptor neprilysin inhibitor; HF, heart failure; CRT, cardiac resynchronization therapy; FU, follow-up; GFR, glomerular filtration rate; HR, hazard ratio; ICD, implantable cardioverter defibrillator; LVEF, left ventricular ejection fraction; MRA, mineralocorticoid antagonist; NA, not available/not applicable.

## 5. Lung Ultrasound During Stress Echocardiography According to Heart Failure Phenotypes

Lung ultrasonography is a reliable and reproducible tool to assess EVLW during stress echocardiography in HF patients (both in HFrEF and in HFpEF,) in conjunction with ESE or pharmacological stressors, both at submaximal and maximal workloads [32, 33, 60, 61, 94]. In a large mixed cohort of 2145 patients referred for stress echocardiography (exercise ~45%, dypiridamole ~50%) with known/suspected coronary artery disease or HF, approximately 15% developed moderate or severe pulmonary congestion as assessed with the 4-zone protocol by Scali *et al*. [61] (see Table 1 for LUS timing and congestion grading during stress echocardiography), while severe stress B-lines (HR 3.54, 95% CI 1.47–8.69, *p* = 0.006) independently predicted long-term death or nonfatal myocardial infarction. The authors concluded that stress echocardiography can be easily complemented with LUS to assess dynamic changes in pulmonary congestion through B-line quantification and better stratify the prognosis of HF patients. These findings confirmed earlier preliminary results from the same group [95].

With particular reference to HF patients, LUS has been shown to enable real-time monitoring of pulmonary congestion elicited by exercise, showing a swift increase in B-line count [96]. Agricola *et al*. [36] first described B-line development during exercise with LUS (28-zone protocol) performed in the recovery phase (>6 minutes after the end of the exercise phase) in a cohort of 72 HF patients referred for exercise echocardiography (approx. 75% with a LVEF <40%). B-line score increased significantly with exercise (5.9 ± 14.9 versus 11.0 ± 20.7,* p* = 0.0001), and its variation was correlated with changes in estimated pulmonary capillary wedge pressure (PCWP), pulmonary artery systolic pressure (PASP) and wall motion score index, and with peak E/e’. B-line development during exercise can also represent a useful risk stratifier in HF outpatients. The prognostic value of exercise LUS in HFrEF patients was first demonstrated by Scali *et al*. [33] in a cohort of 103 HFrEF patients (LVEF <45%) undergoing maximal semi-supine bicycle ESE, with a 28-zone protocol performed at the end of exercise. Median B-line count increased from 5 to 12, and stress B-lines showed good correlation with baseline natriuretic peptide, as well as with stress E/e’ and stress systolic pulmonary artery pressure [33]. Using ROC analyses, a stress B-line count ≥30 was found as the optimal threshold for predicting mortality at a median follow-up of 8 months (AUC 0.83, sensitivity 100%, specificity 73%), while the addition of stress B-line to clinical parameters, BNP and peak ${\mathrm{VO}}_{2}$ was associated with improved mortality risk classification. These findings were confirmed in another cohort of 105 HFrEF patients which reported similar associations between stress B-line count (assessed with a 28-zone protocol) and the composite endpoint of cardiovascular death or HF hospitalisation during a mean follow-up of 29 months [97].

With respect to HFpEF, we demonstrated that submaximal ESE coupled with LUS (28-zone protocol) allowed the detection of pulmonary congestion development (median B-line count from 3 to 9) in a cohort of 31 HFpEF patients, occurring concomitantly with changes in E/e’, PASP and natriuretic peptides (i.e., BNP); these variations were significantly greater in magnitude when compared with changes observed in the control group (N=19 hypertensive patients) [32]. In a subsequent study, we found that these B-line changes were mostly predicted by worsening echocardiographic indices of diastolic function (i.e., E/e’ and strain rate-derived A wave) in an extended cohort of 81 patients [94]. In another study comprised of a cohort of 61 invasively-proven HFpEF patients undergoing submaximal haemodynamic exercise testing, the onset or increase in B-lines (assessed in 2 positions in the left third intercostal space along the mid-axillary and mid-clavicular lines) was associated with an increase in both PCWP and right atrial pressure, and to an impairment in right ventricular (RV)-to-pulmonary circulation coupling, both at rest and during exercise, as assessed by ratios of tricuspid annular plane systolic excursion (TAPSE) and RV s’ or invasive mean pulmonary arterial pressure [34]. Additionally, in another cohort of 188 HFpEF patients undergoing combined cardiopulmonary-echocardiography exercise stress testing, epicardial adipose tissue accumulation (i.e., >5 mm in parasternal long-axis) was related to both higher peak/change B-lines (as assessed with the 8-zone protocol) and reduced peak TAPSE/PASP ratio [98]. Overall, the development of pulmonary congestion in HFpEF is concomitant to increased pulmonary capillary hydrostatic pressures, left ventricular wall stress, and systemic venous hypertension, the latter being associated with impairments in RV-pulmonary artery pressure coupling.

In the same cohort of HFpEF patients [94], we demonstrated that both peak B-line counts (HR 1.50, 95% CI, 1.21–1.85, *p *<0.001) and their changes (HR 1.34, 95% CI 1.12–1.62, *p* = 0.002) were retained as independent predictors of outcome (composite of cardiovascular death or HF hospitalisation at 1 year), along with BNP and E/e’ ratio. B-line counts assessed with 8 zones also independently predicted outcome. Among tested cut-offs, both peak and B-line change >10 appeared to better stratify prognosis in this cohort. Furthermore, adding peak or B-line change, as well as peak B-line >10, significantly improved prognostic accuracy on top of a clinical model (C-index increase ~0.13 and *p *< 0.04 for all values), with similar results for B-line change [35]. These results were recently confirmed by Pugliese *et al*. [99] in a mixed cohort of 274 patients (161 with HFpEF and 113 with stages A–B HF according to the American Classification) undergoing symptom-limited cardiopulmonary exercise testing-exercise stress test echocardiography. Following multivariable analyses, B-line change >10 (assessed with the 8-zone protocol) was retained as an independent predictor of cardiovascular death or HF hospitalisation at long-term, along with peak ${\mathrm{VO}}_{2}$ <16 mL/kg/min, minute ventilation/carbon dioxide production slope >36, PASP >50 mmHg, and resting NT-proBNP >900 pg/mL. Among these predictors, delta B-lines >10 displayed the highest association with the combined endpoint [99].

## 6. Conclusions

LUS represents a reliable and useful tool for the assessment of pulmonary congestion and risk stratification of HF patients throughout the entire patient journey (i.e., ED/acute settings, in-hospital management, discharge from an acute HF hospitalisation, and monitoring in the outpatient setting), with considerable diagnostic and prognostic implications. The 8-zone protocol appears to offer the best trade-off with no sizeable loss of information.
